# Musculoskeletal Injury Risk Stratification: A Traffic Light System for Military Service Members

**DOI:** 10.3390/healthcare11121675

**Published:** 2023-06-07

**Authors:** Megan H. Roach, Matthew B. Bird, Matthew S. Helton, Timothy C. Mauntel

**Affiliations:** 1 Extremity Trauma & Amputation Center of Excellence, Defense Health Agency, Falls Church, VA 22041, USA; matthew.b.bird.civ@health.mil (M.B.B.); timothy.c.mauntel.civ@health.mil (T.C.M.); 2 Department of Surgery, Uniformed Services University of the Health Sciences, Bethesda, MD 20814, USA; 3 Department of Clinical Investigations, Womack Army Medical Center, Fort Liberty, NC 28310, USA; 4 U.S. Army, Fort Liberty, NC 28310, USA; matthew.s.helton2.mil@army.mil

**Keywords:** injury mitigation, movement screen, risk factor

## Abstract

Risk factor identification is a critical first step in informing musculoskeletal injury (MSKI) risk mitigation strategies. This investigation aimed to determine if a self-reported MSKI risk assessment can accurately identify military service members at greater MSKI risk and determine whether a traffic light model can differentiate service members’ MSKI risks. A retrospective cohort study was conducted using existing self-reported MSKI risk assessment data and MSKI data from the Military Health System. A total of 2520 military service members (2219 males: age 23.49 ± 5.17 y, BMI 25.11 ± 2.94 kg/m^2^; and 301 females: age 24.23 ± 5.85 y, BMI 25.59 ± 3.20 kg/m^2^, respectively) completed the MSKI risk assessment during in-processing. The risk assessment consisted of 16 self-report items regarding demographics, general health, physical fitness, and pain experienced during movement screens. These 16 data points were converted to 11 variables of interest. For each variable, service members were dichotomized as at risk or not at risk. Nine of the 11 variables were associated with a greater MSKI risk and were thus considered as risk factors for the traffic light model. Each traffic light model included three color codes (i.e., green, amber, and red) to designate risk (i.e., low, moderate, and high). Four traffic light models were generated to examine the risk and overall precision of different cut-off values for the amber and red categories. In all four models, service members categorized as amber [hazard ratio (HR) = 1.38–1.70] or red (HR = 2.67–5.82) were at a greater MSKI risk. The traffic light model may help prioritize service members who require individualized orthopedic care and MSKI risk mitigation plans.

## 1. Introduction

Musculoskeletal injuries (MSKIs) compromise service member health, wellness, and readiness, and thus pose a significant public health burden for the military [1,2]. Non-battle related MSKIs affect 63% of non-deployed military personnel and account for 90% of all injuries among deployed service members [3,4]. Collectively, non-battle-related MSKIs account for up to 60% of limited duty days and roughly $548 million in direct patient care costs each year [5,6]. Consequently, there is a significant need to develop MSKI risk identification models and mitigation strategies to maintain readiness for military force protection worldwide.

Multifactorial MSKI risk identification models are required to effectively identify service members’ at an increased MSKI risk [7,8,9]. These multifactorial MSKI risk identification models commonly include health questionnaires, physical performance measures, clinical assessments, and movement screenings; recently, commercial technologies have been used to expedite and make MSKI risk assessments more objective [7,8,9,10,11]. A meta-analysis found more than 950 MSKI risk factor variables were identified across 170 studies published between 1995 and 2020 [12]. Of those variables, there was strong scientific evidence for female gender, high body mass index (BMI), low physical fitness, prior MSKI, and pain with Functional Movement Screen (FMS) tests as MSKI risk factors [12,13,14]. While self-reported questionnaires may not provide the same level of detail as sophisticated measurements, all of these variables can be captured via questionnaires with minimal effort and time. An efficient, comprehensive self-report MSKI risk assessment has not been used as of yet to build an MSKI risk identification model for service members.

MSKI risk identification models use multiple variables to calculate the probability of a health outcome (e.g., MSKI) [15]. A variety of approaches have identified MSKI risks in service members [8,9,16,17,18]. One approach is to use robust advanced predictive analytics [10,19,20], such as machine learning, to develop MSKI prediction models. While this type of approach typically yields better performances [18], the advanced predictive models require extensive time and expertise, making the clinical translation and implementation difficult and often unrealistic in the current military environments. Another approach is to establish clinically informed and relevant thresholds (e.g., >4 risk factors) to identify service members at a higher risk for MSKI. This approach (i.e., summation of the number of risk factors), produced highly sensitive and specific MSKI risk models in U.S. Army Rangers; MSKI risk increased for Rangers when four or more risk factors were present [8]. However, a shortcoming of this approach is that too many service members may be categorized as high risk, and medical manpower may not be available to provide appropriate assessments and mitigation strategies. A traffic light model may therefore offer a simple solution to triage service members as low (green), moderate (amber), and high risk (red) for MSKI. The traffic light model will optimize the use of medical assets by targeting service members with the highest MSKI risk.

Risk factor identification is a critical first step in informing MSKI mitigation strategies and retaining mission-ready service members. However, the ability of a comprehensive, self-report MSKI risk assessment to correctly identify the service members who are at a greater risk for MSKI is unknown. Furthermore, despite significant progress in understanding the characteristics associated with future MSKI risk, a clinical decision support tool (e.g., traffic light model) to screen service members’ MSKI risk has not been developed. Therefore, the purpose of this study was as follows: (1) to determine if items on a self-report MSKI risk assessment can accurately identify service members at a greater MSKI risk, and (2) determine if a traffic light model to identify MSKI risk-level can differentiate service members’ risks of sustaining MSKIs. We hypothesized that previously identified MSKI risk factors (e.g., prior MSKI, prior surgery, profile for MSKI, slower 2-mile run, less sleep, depression, nicotine use, and pain during movement) would increase MSKI risk. Furthermore, due to the potential overlap with prior MSKI risk factors, we hypothesized that stress fracture history, pain during a physical fitness test, and failing a physical fitness test would increase MSKI risk. We also hypothesized that service members categorized as amber (moderate risk) or red (high risk) in the traffic light model would be at a higher risk for MSKI than service members categorized as green (low risk).

## 2. Materials and Methods

### 2.1. Study Design & Setting

We conducted a retrospective cohort study in a U.S. Army division located at Fort Liberty NC (formerly Fort Bragg), utilizing data from a self-report MSKI risk assessment and MSKI data from the Military Health System Management Analysis and Reporting Tool (MHS MART [M2]). The cohort included all active-duty service members in-processing to the 82nd Airborne Division at Fort Liberty between December 2020 and July 2021. This investigation received an exempt determination as non-human subject research by the local Human Research Protections Office.

### 2.2. Self-Report Musculoskeletal Injury Risk Assessment

Service members completed a self-report MSKI risk assessment as part of their in-processing requirements. Service members were instructed to scan a QR code linked to the questionnaire and complete the questionnaire on their phones. The self-report format and ability for multiple service members to complete the screening simultaneously allowed for up to 150 service members to be screened in less than 30 min. The questionnaire consisted of 16 self-report items regarding demographics, general health, physical fitness test performance, and pain or stiffness experienced during movement screens (Table 1). Movement screens included the Functional Movement Screen (FMS), shoulder clearing (bilaterally) and spinal extension clearing tests [21], and a squat-jump-and-land. For the squat-jump-and-land service members were instructed to squat, jump, and land in an upright position with the legs at a self-selected width. Cadre from the in-processing team led service members through each movement screen. After each movement screen, service members annotated whether they experienced pain or stiffness on the questionnaire; no additional information was recorded about the movement screen by the service member or cadre. The questionnaire items and movement screens were selected based on clinician expertise and the best available research evidence pertaining to MSKI risk factor identification in light infantry airborne service members [8,9].

### 2.3. Musculoskeletal Injury Data

MHS MART is a centralized data repository that captures and catalogs data input into the Military Health System’s electronic medical records. MSKI data, including the encounter date and the tenth edition International Classification of Diseases (ICD-10) codes, were extracted from the Comprehensive Ambulatory Provider Encounter Records (CAPER) within M2. The CAPER was searched for MSKI encounters from the service member’s MSKI risk assessment date to 1 December 2022. The ICD-10 codes corresponding to MSKIs were selected based on prior MSKI taxonomy [22,23], and identified service members who sustained an MSKI within the first year following their in-processing screen. Gender and age data were also extracted from M2.

### 2.4. Data Reduction

The 16 self-report MSKI risk assessment data points were converted into 11 variables of interest. The items pertaining to height and weight were removed to calculate and control for BMI, and the four movement screen items were combined into a single variable. For the 11 variables of interest, service members were dichotomized as at risk or not at risk (Table 1). For categorical variables, a “yes” response was considered at risk and a “no” response not at risk. Stiffness reported during the movement screens was annotated as “no pain” for analyzes. For continuous variables, responses were categorized as at risk and not at risk, respectively, based on established cut-points. The cut-point (<90 points) for an average 2-mile run time was based on the Army Combat Fitness Test (ACFT) age and gender standards [24]. The cut-point for average sleep duration was six hours based on previous literature indicating U.S. Army soldiers with an MSKI reported sleeping less per night than uninjured soldiers (5.7 h vs 6.1 h) [25]. M2 MSKI data were cleaned to isolate the initial MSKI encounter, and all subsequent MSKI encounters were then removed from the dataset. Survival time was calculated as time from the self-report MSKI risk assessment date to the first MSKI encounter date for injured service members, and time from MSKI risk assessment date to the end of the surveillance period (up to 365 days) for uninjured service members. For this analysis, we did not differentiate between the service members with one versus more than one MSKI or evaluate how the risk factors influenced subsequent MSKI risk.

### 2.5. Data Analyses

All data analyzes were performed using R Statistical Software version 4.4.2 and packages including, riskRegression, tidyverse, survival, and survminer [26]. Descriptive statistics were calculated to describe the cohort, risk factors, and traffic light models as appropriate.

Cox proportional hazard regression models, adjusted for age, gender, and BMI, estimated hazard ratios (HRs) and 95% confidence intervals (CIs) to determine which MSKI risk assessment variables of interest were associated with a greater MSKI risk during the surveillance period. Statistically significant variables (*p* < 0.05) from the adjusted models were considered as risk factors and were used to develop the four traffic light models. Prior to entry into the traffic light models, multicollinearity was evaluated with variance inflation factor (VIF) calculations; risk factors with a VIF > 10 were subjected for removal.

Each traffic light model included three color codes (i.e., green, amber, and red) to designate MSKI risk level (i.e., low, moderate, and high). To examine the risk and overall model performance of different cut-off values for the moderate and high-risk groups, four separate traffic light models were generated. Service members were stratified based on the number of risk factors identified on the self-reported MSKI risk assessment. Service members with no risk factors were always categorized as green (i.e., low risk), and the threshold for a service member to be categorized as amber (i.e., moderate risk) or red (i.e., high risk) increased by one risk factor in each traffic light model which was as follows: *Model I*: green = 0, amber = 1–2, red = ≥ 3; *Model II*: green = 0, amber = 1–3, red = ≥ 4; *Model III*: green = 0, amber = 1–4, red = ≥ 5; and *Model IV*: green = 0, amber = 1–5, red = ≥ 6. Kaplan–Meier survival curves with median survival times were generated to visualize risk profiles by color for each traffic light model. The median survival time represents the time at which the survival probability is 0.50 for each traffic light color (i.e., half of the service members remain MSKI free) [27]. Unadjusted and adjusted hazard ratios with 95% CIs were also calculated for each traffic light model to determine if the traffic light categories could differentiate the service members at risk of sustaining an MSKI during the surveillance period (*p* < 0.05). The time-dependent area under the curve (AUC) and standard error values were calculated at 30, 60, 90, 180, and 365 days, respectively, with 10 bootstrap cross validations at each time point to analyze the ability of each traffic light model to identify MSKI risk over the one-year surveillance period. AUC performance was interpreted as by chance (0.50–0.60), poor (0.61–0.70), fair (0.71–0.80), good (0.81–0.90), and excellent (0.91–1.00) [28,29].

## 3. Results

### 3.1. Service Members

A total of 2520 service members (2219 males: age 23.49 ± 5.17 y, BMI 25.11 ± 2.94 kg/m^2^; and 301 females: age 24.23 ± 5.85 y, BMI 25.59 ± 3.20 kg/m^2^) completed the MSKI risk assessment during in-processing. Within the cohort, 1349 (53.5%) service members had an MSKI documented in their medical record during the surveillance period. Of the first MSKI medical encounters used for this analysis, the majority were for the lower extremity (51.2%), followed by the spine and back (19.9%). Less than half of the cohort (40.56%) reported no risk factors on the self-reported MSKI risk assessment. Nicotine use was the most frequent risk factor reported at 31.7%. (Table 1).

### 3.2. Musculoskeletal Risk Assessment Variables of Interest

In Cox proportional regression models adjusted to age, gender, and BMI, the MSKI rate during the surveillance period was found to be greater in service members with an MSKI in the last year, surgery in the last two years, a limited duty profile >3 days for MSKI in the last year, a history of stress fracture, pain during the last physical fitness test, slower 2-mile run times, less sleep, feeling down, depressed, or hopeless, and pain on a movement screen (Table 2). Failing a physical fitness test event in the last year and nicotine use were not found to be independent risk factors when adjusted for age, gender, and BMI. Thus, nine of the eleven variables of interests were considered as risk factors for MSKI and were used to build four separate traffic light models based on different risk factor thresholds for the amber and red categories. VIFs for all risk factors were <10 (VIF range: 1.06–1.91), indicating no multicollinearity.

### 3.3. Musculoskeletal Injury Risk Traffic Light Model

**Figure 1:**
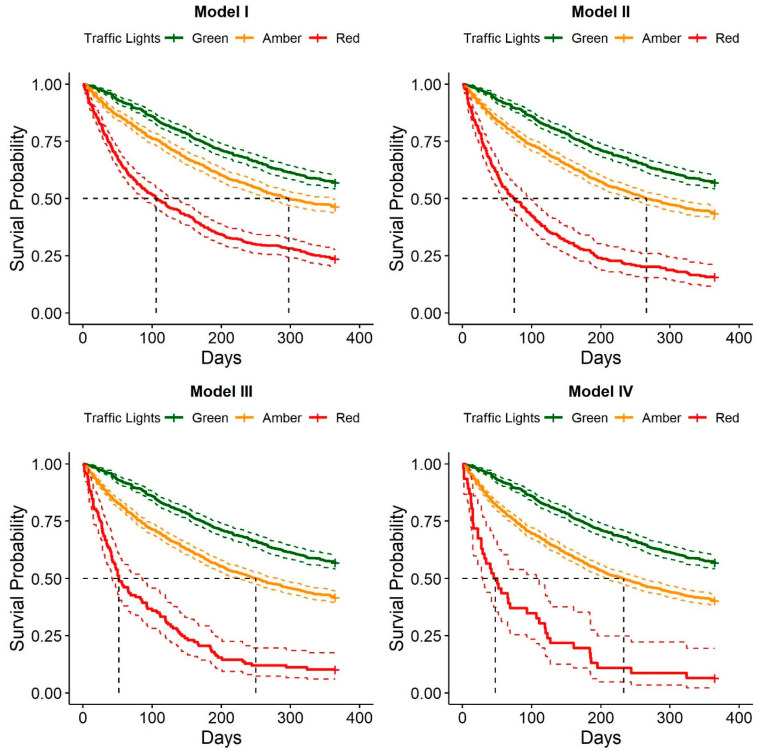
Kaplan–Meier survival probability by risk category *Models I*, *II*, *III*, and *IV*. The CI for each survival curve is plotted (green, amber, red). The black dashed lines represent the median survival probability.

**Figure 2:**
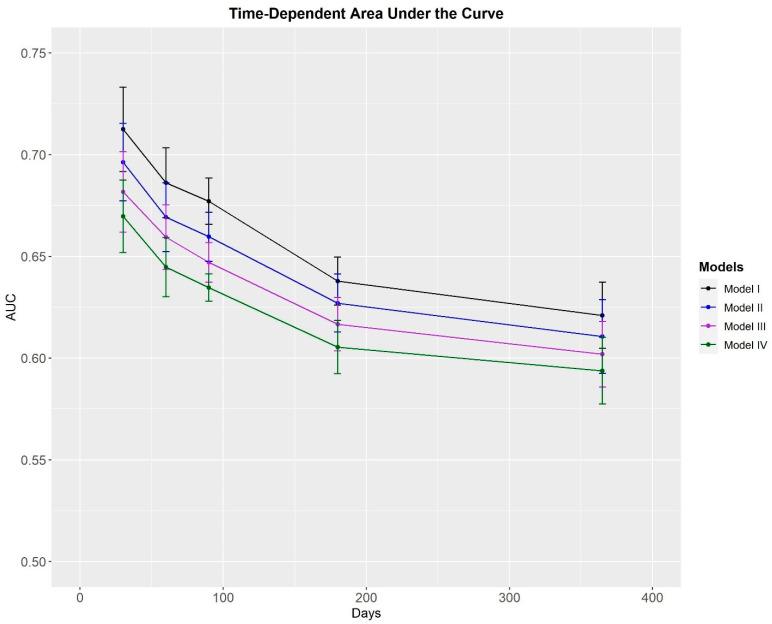
Area under the curve values plotted over the surveillance period for the unadjusted traffic light models.

**Figure 3:**
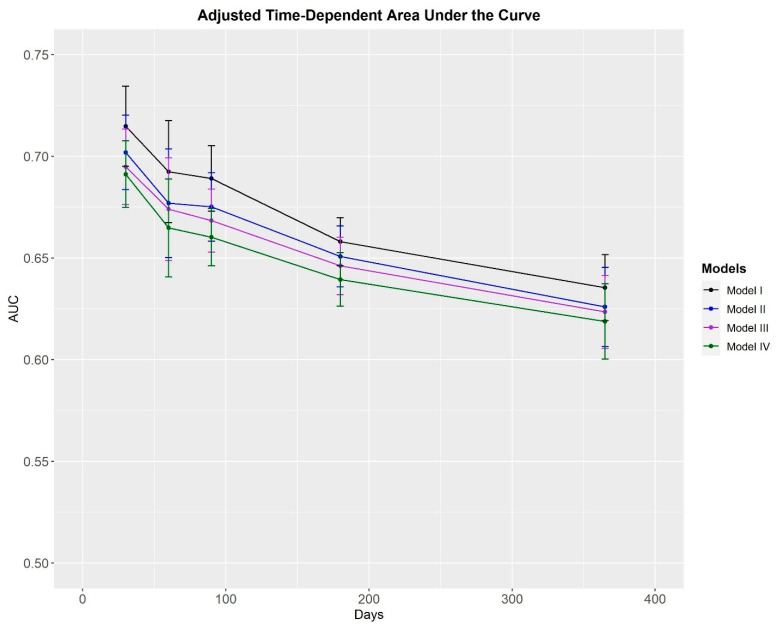
Area under the curve values plotted over the surveillance period for the adjusted traffic light models.

In all four traffic light models, median survival probability was found to be greatest for the service members who were categorized as green (Figure 1). In the unadjusted and adjusted traffic light models, the MSKI risk was greater in service members categorized as amber (HR = 1.38–1.70) and Red (HR = 2.67–5.82) compared to service members categorized as green (Table 3). Time-dependent AUC performance declined over the surveillance period in the unadjusted (Figure 2) and adjusted (Figure 3) traffic light models. Adjusted and unadjusted *Model I* (Red = ≥ 3 risk factors) outperformed *Models II*, *III*, and *IV* across all time points, with AUC values ranging from fair (AUC = 0.71) at 30 days to poor (AUC = 0.62–0.64) at 365 days.

## 4. Discussion

The results of this retrospective cohort study indicate a comprehensive self-report MSKI risk assessment can accurately identify service members at greater MSKI risk. This is the first study to examine the utility of an MSKI risk assessment based entirely on self-reported responses, collected administratively by a U.S. Army division. Prior military MSKI risk assessments have used a combination of physical tests, functional movement screens, and self-reported information collected by highly trained research teams [8,9]. This study also provides a traffic light model to tier MSKI risk (i.e., low, moderate, and high). As hypothesized, service members categorized as amber and red in the traffic light model were found to be at a greater risk for MSKI than service members categorized as green. These findings suggest that a self-reported MSKI risk assessment can identify the service members at a greater MSKI risk, and a single data point (green, amber, or red) may assist medical assets with identifying service members for individual evaluation and MSKI risk mitigation plans.

Of the eleven variables of interest, nine were considered risk factors, the majority of which have been previously linked to MSKI risk [8,9,30]. In agreement with the previous literature, surgery in the last two years, MSKI within the last year, and pain on a movement screen test placed service members at twice the risk for MSKI during the surveillance period [8,9]. To our knowledge, this is the first military MSKI risk assessment to include pain during the last physical fitness test (1.90 times greater risk) and feeling down, depressed, or hopeless (1.46 times greater risk) as independent MSKI risk factors. Stress fracture history and pain during the last physical fitness test were hypothesized as risk factors as stress fracture history overlaps with prior MSKI, and pain experienced during a physical fitness test involving complex movements is likely associated with pain during movement assessments (i.e., clearing tests). It is important to note the questionnaire items used in the current study asked about MSKI in the last year and any history of stress fracture (i.e., lifetime), thus potentially explaining the low observed VIF values (<1.91), representing a low multicollinearity between the variables. Similarly, depression was hypothesized to be a risk factor, as depressive symptoms, including a lack of concentration and daytime drowsiness, are risk factors for unintentional injury [31].

Contrary to our hypotheses, nicotine use and failing a physical fitness test (i.e., <70% on any event) were not independent MSKI risk factors. These findings are also contradictory to previously reported MSKI risk factors [12,14]. However, nearly a third of the population used nicotine (31.7%), and a fifth of the population failed a physical fitness assessment in the past year (18.2%). Thus, these potential risk factors may not be unique enough to be significant factors in the current cohort. Service members could have failed any of the three Army Physical Fitness Test (APFT: push-ups, sit-ups, and 2-mile run time) or six ACFT (three-repetition maximum deadlift, standing power throw, hand-release push-up, sprint-drag-carry, leg tuck, and 2-mile run time) events in the last year. We do not know which of the physical fitness assessment tests the service members have failed in the past year, and thus we do not have the granularity to individually evaluate each assessment as a risk factor, as previous studies have conducted [32,33,34]. Had we been able to individually evaluate each component of the physical fitness assessments, one or more of the individual events may have been identified as an MSKI risk factor. However, the wide range of risk factors identified in the current investigation support the use of multifactorial MSKI risk identification strategies and suggest a self-report MSKI risk assessment implemented within an operational unit is equally capable of identifying MSKI risk factors as MSKI risk assessments used in research settings.

Secondary to identifying self-report MSKI risk factors, we aimed to determine whether a clinical decision support tool based on a traffic light model would be capable of discriminating between service members categorized as green, amber, and red, respectively. In adjusted and unadjusted models, service members categorized as amber and red were at a higher MSKI risk than those categorized as green. Our study also confirmed prior findings that as the number of risk factors present increased, the MSKI risk also increased as a result [9]. As we increased the risk factor threshold for entry into the red (i.e., high risk) category across traffic light models the HRs steadily increased. Based on the adjusted models, service members categorized as red with ≥3 risk factors (*Model I*) were at 2.67 times the risk of MSKI, whereas those categorized as red with ≥6 risk factors (*Model IV)* were at 4.52 times the risk of MSKI compared to service members categorized as green. A similar trend was observed for service members categorized as amber; service members with one or two risk factors (*Model I*) were at 1.38 times the risk of MSKI, whereas those with one to five risk factors (*Model IV*) were at 1.63 times the risk of MSKI. Thus, the traffic light models, regardless of risk factor threshold were able to differentiate between the color categories.

Of equal importance, the median survival time (i.e., time of remaining MSKI free) decreased as the number of reported risk factors increased. The median survival time for the amber and red categories ranged from 233 to 298 days and 48 to 106 days, respectively, across all four traffic light models, with an inverse relationship observed between the number of risk factors present and the time of remaining MSKI free (i.e., the more risk factors present, the earlier a service member would report an MSKI). This is important, as one of the primary risk factors for future MSKI is prior MSKI [8,9]. Thus, theoretically, the earlier a service member sustains an MSKI, the more likely they will sustain a subsequent MSKI. Collectively, this pattern of MSKI and subsequent MSKIs can significantly diminish the service member’s physical abilities, medical readiness, and ability to perform their job.

Prior to implementing an MSKI risk identification model, the model should be able to discriminate between the individuals with and without MSKI. One way to measure its discriminatory ability is with an AUC value [35,36]. AUC values > 0.7 demonstrate an acceptable discriminatory ability [37], suggesting a clinically meaningful ability to identify individuals at an increased risk of incurring a MSKI. Our findings indicate the traffic light models were able to discriminate between the service members with and without a MSKI. In *Model I*, the adjusted AUC values (Figure 3) demonstrated a *fair* (30-days = 0.71) to *poor* (365-Day = 0.64) performance, gradually decreasing over time. Similar trends were observed for *Model II* (30-Days = 0.70; 365-Days = 0.63), *Model III* (30-Days = 0.69; 365-Days = 0.62), and *Model IV* (30-Days = 0.69; 365-Days = 0.62) and for the unadjusted models (Figure 2). All traffic light models performed the best at the 30-day mark, suggesting the discriminatory ability of this type of model degrades over time. The degradation in model performance was likely the result of fewer service members available to discriminate between towards the end of the surveillance period. The decrease in discriminatory ability could also be attributed to the variability within the modifiable risk factors. Slightly more than half of the nine risk factors (pain on a movement screen test; pain during the last physical fitness test; feeling depressed; slower 2-mile run time; and less sleep) included on the MSKI risk assessment were modifiable. Thus, whether the service member’s response is categorized as at risk or not at risk could alter week-to-week or month-to-month, thereby influencing model performance. Furthermore, all four of the non-modifiable risk factors (surgery in the last two years; MSKI within the last year; limited duty profile >3 days for MSKI in the last year; and stress fracture history) included in our traffic light models relate to prior MSKI, and most involved a time element (i.e., in the last year). Thus, accounting for prior MSKIs and prior MSKI surgery beyond the timeframes specified, as well as time since MSKI or surgery in future work may retain model performance over time.

Across traffic light models, the proportion of service members categorized as red gradually decreased as the risk factor threshold increased. *Models I, II, III,* and *IV* categorized roughly 18%, 9%, 5%, and 2% of the cohort as red, respectively. This type of adjustment offers medical personnel the opportunity to tailor traffic light models to meet their unique needs and bandwidth to provide more personalized care. Providing further evaluation and MSKI mitigation plans may be more feasible for 46 or 117 service members (*Model IV* and *III*) versus 229 or 466 service members (*Models I* and *II*), respectively. Thus, if medical resources are limited, the risk factor threshold can be increased to prioritize service members at the greatest risk for MSKI. This type of flexible tiered system adds to the clinical utility of the traffic light model for MSKI risk identification. However, clinicians should be cognizant of model performance for discriminating between the service members with low, moderate, and high MSKI risks (i.e., AUC), as it decreases as the amber and red group thresholds increase.

The self-report MSKI risk assessment and traffic light model as a MSKI risk identification strategy may improve the overall efficiency of medical systems. The self-report MSKI risk assessment is expeditious and cost-effective. The 16-item questionnaire does not require the extensive time, equipment, or manpower, as some of the physical assessments and commercial technologies (e.g., force plates, physiological monitors, and 3-D motion capture) used to identify individuals at risk for MSKI [10,22]. More notably, the data collected can be analyzed and interpreted without a data scientist, complex algorithms, or expensive software. Although our model did not perform as well as prior derived models (AUC values = 0.89–0.90) with continuous predictors and risk factor selection based on rationale and clinical reasoning [18], our models adjusted for age, gender, and BMI demonstrated better discrimination (AUC values = 0.64–0.71) across the 1-year surveillance period than a prior military MSKI risk identification model (AUC = 0.63) that dichotomized risk factors [18]. The prior-derived models [18] did not account for time since screening, meaning it is possible that model performance may have decreased similar to what was observed in the current investigation. While the discriminatory capabilities of the traffic light MSKI risk identification strategy are only *poor* to *fair*, the more pragmatic approach of the traffic light system may offset its inferior ability to discriminate between service members at low vs high risk of MSKI.

### Limitations

The self-report MSKI risk assessment and traffic light models provide some clinical utility for MSKI risk identification, particularly within the first 30-days. However, limitations should be considered when interpreting the results of the current investigation. First, the average sleep and 2-mile run times were entered into the traffic light model as dichotomized variables. This approach decreases fidelity [38,39,40], but may help with interpretation and clinical feasibility. Second, we did not weight risk factors in this investigation. It has been well-established that some MSKI risk factors are more predictive than others in military populations [14]. Additionally, some risk factors can be modified whereas others are non-modifiable [14]. Thus, incorporating a weighted element for each MSKI risk factor may help improve the model performance. Third, we were unable to determine injury severity, mechanism of injury, or the number of days a service member did not participate in physical activity after they sustained an MSKI; thus, we could not account for these factors in our analyzes. Fourth, military occupational specialty may influence MSKI risk, however we were unable to adjust for this variable in our models. Lastly, the self-reported MSKI risk assessment data were only collected in a single U.S. Army division, potentially limiting the generalizability of our findings. Future work will seek to externally validate our traffic light model, evaluate how weighting risk factors influences model performance, and explore how these risk factors relate to recurrent and subsequent MSKIs.

## 5. Conclusions

Service members who self-report more MSKI risk factors are more likely to sustain an MSKI. More specifically, service members categorized as red (i.e., high risk) in the traffic light models were up to 4.5 times more likely to incur an MSKI as compared to service members categorized as green (i.e., low risk). The traffic light model may help improve the clinical utility and interpretation of comprehensive MSKI risk assessments, as clinicians and military leaders are provided with a single data point (i.e., green, amber, or red) to help inform them as to who requires individualized evaluation and care. This approach is time- and resource-efficient and provides an easy-to-interpret data point that can be utilized across medical and performance specialties, resulting in holistic and more personalized MSKI risk mitigation programs, culminating in fewer MSKIs and decreased limited duty time, thereby improving force medical readiness.

## Figures and Tables

**Table 1:** Self-reported musculoskeletal injury risk assessment.

Item	At Risk Response	At Risk %	Not at Risk Response	Not at Risk %
Have you had a musculoskeletal injury within the last year which required medical attention or lasted more than 1-week in duration?	Yes	16.7%	No	83.3%
Have you had a surgery in the last 2-years that required Physical Therapy?	Yes	1.7%	No	98.3%
Have you been placed on a temporary profile for more than 3-days in the last year for a musculoskeletal injury?	Yes	13.2%	No	86.8%
Have you ever been diagnosed with a stress fracture?	Yes	6.1%	No	93.9%
During your last APFT/ACFT did you have pain?	Yes	16.1%	No	83.9%
What is your height in inches? ^#^	---	---	---	---
What is your weight in pounds? ^#^	---	---	---	---
What is your average 2-mile run time?	<90 Points	24.4%	≥90 Points	75.6%
Have you scored less than 70% on any APFT/ACFT event in the last year?	Yes	18.2%	No	81.8%
How much sleep do you get on average?	<6 h	18.7%	≥6 h	81.3%
Are you often bothered by feeling down, depressed, or hopeless; OR bothered by little interest or pleasure in doing things?	Yes	8.1%	No	91.9%
Do you use nicotine at all?	Yes	31.7%	No	68.3%
Did you have pain or experience stiffness with the movement?	Experienced pain	21.9%	No pain	78.1%
Right shoulder clearing test				
Left shoulder clearing test				
Spinal extension clearing test				
Squat-jump-and-land				

Abbreviations: ACFT, army combat fitness test; and APFT, army physical fitness test. ^#^ Converted to body mass index for use in the adjusted models.

**Table 2:** Adjusted hazard ratios for the variables of interest.

Risk Factor	HR	95% CI	*p*-Value
Surgery in the last two years that required physical therapy	2.40	1.72–3.34	<0.001
Musculoskeletal injury within the last year	2.05	1.80–2.33	<0.001
Pain on a movement screen test	2.01	1.78–2.26	<0.001
Limited duty profile for musculoskeletal injury in the last year	1.99	1.73–2.29	<0.001
Pain during the last physical fitness test	1.90	1.67–2.17	<0.001
Feeling down, depressed, or hopeless	1.46	1.22–1.74	<0.001
Stress fracture history	1.45	1.18–1.77	<0.001
Slower 2-mile run time (*<90 Points Based on Age & Gender*)	1.33	1.17–1.50	<0.001
Less sleep (*<6 h*)	1.21	1.06–1.38	0.005
Failing a physical fitness event in the last year	1.12	0.98–1.29	0.097
Nicotine use	1.04	0.93–1.17	0.495

Note: all hazard ratios were adjusted for age, gender, and body mass index. Abbreviations: CI, confidence interval; and HR, hazard ratio.

**Table 3:** Cox proportional regression models for each traffic light model.

Traffic Light Model	Traffic Light Color	Risk Factors Present	*n*	Proportion of Population	Median Survival Time (Days)	Unadjusted HR (95% CI)	Unadjusted *p*-Value	Adjusted HR (95% CI)	Adjusted *p*-Value
I	Green	0	1022	40.56%	--	--	--	--	--
	Amber	1–2	1032	40.95%	298	1.40 (1.24–1.60)	<0.001	1.38 (1.21–1.56)	<0.001
	Red	≥3	466	18.49%	106	2.91 (2.53–3.35)	<0.001	2.67 (2.31–3.09)	<0.001
II	Green	0	1022	40.56%	---	---	---	---	---
	Amber	1–3	1269	50.36%	266	1.54 (1.37–1.73)	<0.001	1.49 (1.32–1.68)	<0.001
	Red	≥4	229	9.09%	75	3.90 (3.29–4.63)	<0.001	3.47 (2.91–4.15)	<0.001
III	Green	0	1022	40.56%	---	---	---	---	---
	Amber	1–4	1381	54.80%	250	1.63 (1.45–1.83)	<0.001	1.57 (1.39–1.76)	<0.001
	Red	≥5	117	4.64%	52	5.00 (4.03–6.20	<0.001	4.34 (3.48–5.42)	<0.001
IV	Green	0	1022	40.56%	---	---	---	---	---
	Amber	1–5	1452	57.62%	233	1.70 (1.52–1.91)	<0.001	1.63 (1.45–1.83)	<0.001
	Red	≥6	46	1.83%	48	5.82 (4.25–7.97)	<0.001	4.52 (3.27–6.26)	<0.001

## Data Availability

The datasets generated and analyzed during the current study are not publicly available because of data sharing restrictions on data generated within the United States Department of Defense; however, data may be available from the corresponding author on reasonable request, following approval from all required regulatory bodies.
